# System Based on an Inertial Measurement Unit for Accurate Flight Time Determination in Vertical Jumps

**DOI:** 10.3390/s23136022

**Published:** 2023-06-29

**Authors:** Juan A. Moreno-Pérez, Isidoro Ruiz-García, Ismael Navarro-Marchal, Nuria López-Ruiz, Pablo J. Gómez-López, Alberto J. Palma, Miguel A. Carvajal

**Affiliations:** 1 ECSens, Sport and Health University Research Institute (iMUDS), Department of Electronics and Computer Technology, ETSIIT, University of Granada, 18014 Granada, Spain; juanantoniomp@ugr.es (J.A.M.-P.); isirg@ugr.es (I.R.-G.); nurilr@ugr.es (N.L.-R.); ajpalma@ugr.es (A.J.P.); 2 SkiingLab, Sport and Health University Research Institute (iMUDS), Department of Physical and Sport Education, University of Granada, 18007 Granada, Spain; ismaelnavarro71@gmail.com (I.N.-M.); pjgomez@ugr.es (P.J.G.-L.); 3 Human Lab, Sport and Health University Research Institute (iMUDS), Department of Physical Education and Sport, Faculty of Sport Sciences, University of Granada, 18007 Granada, Spain

**Keywords:** inertial measurement system, jump monitoring, smartphone app

## Abstract

The world of elite sports has always been characterized by intense competition, where victories are often determined by minimal differences. This means that every little detail in the preparation of top-level athletes is crucial to their performance at the highest level. One of the most significant aspects to monitor is the jumping capacity, as it enables the measurement of performance, progression, and helps prevent injuries. Herein, we present the development of a system capable of measuring the flight time and height reached by the user, reporting the results through a smartphone using an Android ad-hoc application, which handles all the data processing. The system consists of an affordable and portable circuit based on an accelerometer. It communicates with the smartphone via UART using a Bluetooth module, and its battery provides approximately 9 h of autonomy, making it suitable for outdoor operations. To evaluate the system’s precision, we conducted performance tests (counter-movement jumps) with seven subjects. The results confirmed the system’s potential for monitoring high-level sports training sessions, as the average deviation obtained was only 2.1% (~0.01 s) in the analysis of flight time and 4.6% (~0.01 m) in jump height.

## 1. Introduction

Inertial measurement units (IMUs) are currently experiencing a significant surge in popularity due to their application in various sports [1,2,3] and studies involving movement and posture [4,5,6,7,8]. These devices not only provide basic measurements, but also enable the recognition of activities through the processing of the acquired data [9,10,11], often leveraging large databases and employing artificial intelligence techniques.

In the realm of sports, an increasing number of elite athletes and coaches are demanding the use of new technologies to track and enhance their performance, since the significance of even the smallest detail in their techniques can greatly impact their sporting success. In certain sports, victory can be determined by minute differences, making it essential to gather as much information as possible from training sessions to identify areas for improvement. Specifically, jumping is one of the most employed movements in sports, as well as one of the best performance tests [12]. It is used in individual disciplines such as athletics or alpine skiing [13], as well as in team sports like basketball [14,15] or volleyball [16]. In addition, jumping performance is directly related to the lower-body strength [17,18], anaerobic power, and capacity [19].

Some of the measurement systems currently available on the market for this purpose are quite accurate. For instance, force platforms [20,21,22,23] are used by researchers to estimate the height or time-of-flight (TOF) of a vertical jump. These platforms have been extensively validated and proven to be reliable and feasible, serving as a gold standard for validating other measurement systems. Examples of such systems include those based on high-speed cameras and video analysis software [24,25], 3D motion capture [26], photogrammetry [27], optoelectronics systems [28], pressure sensors [29], and photocell mats [30], in which high-speed motion capture systems (HSC) and laser platforms (SJS) are used. In recent years, there has been significant development in inertial systems [31,32], with the aim of maximizing parameterization of training and techniques [33,34,35,36,37,38,39], even using the integrated accelerometer in smartphones [40]. All these systems have been focused on achieving optimal progression and improvement, enabling continuous monitoring of the athlete’s performance and reducing the risk of injury [41]. However, achieving high-accuracy data requires post-processing and/or a laboratory environment.

In the present work, we present an instrumentation system based on a three-axis accelerometer. Together with a custom-made ad-hoc smartphone application, this system provides an immediate measurement of the TOF and the jump height without requiring any additional instrumentation.

## 2. Materials and Methods

### 2.1. Experimental Setup

Seven subjects without gait impairments, one female and six males, aged between 18 and 46 (28 ± 9) participated in this study, whose average weight was (72 ± 9) kg. All subjects were healthy and physically active, as they engaged in more than 3 exercise sessions per week; however, none of them were performing at an elite level. Each subject required approximately 20 min to complete the test, including the test explanation, system preparation and calibration, data collection, and device removal. Prior to the study, written informed consent was obtained from all participants, and the protocol adhered to the standard requirements in sports and exercise science research [42].

The selected system used as the gold standard in this study was the Kistler 9260AA6 (Kistler, Barcelona, Spain), a portable triaxial platform for footprint and balance analysis. Its dimensions are 600 × 500 × 50 mm, and it is capable of measuring forces up to 5 kN. The system exhibits a low linearity error (<±0.5% FSO, Full Scale Output) and hysteresis (<0.5% FSO), as well as high sensitivity (0.9–19 mV/N on the z-axis). The sampling frequency on the z-axis is 250 Hz, which is enough for this application [43]. The platform is connected to the computer via USB through the Bioware software (© 2022 Kistler Group, Barcelona, Spain) to initiate and stop recording the jump sessions, edit the measurements, and download the data for further processing.

Trials were conducted at iMUDS (Instituto Mixto Universitario de Deporte y Salud) of the University of Granada to determine the take-off and landing instants of the jumps from the data provided by the system software. The technique performed by the participants was the countermovement jump (CMJ) [44,45,46], wherein they stood with a knee angle of 180° with their hands on their hips. A countermovement was executed until the knee angle reached approximately 90°, followed immediately by a vertical jump to attain maximum height, landing with the knees still extended at a 180° angle. Each of the seven subjects performed three jumps, resulting in a total of 21 measurements. Data collection was conducted simultaneously using both the force platform and the inertial system, with the latter capable of sending the data to a computer or smartphone via Bluetooth. Measurements started a couple of seconds prior to each subject initiating the jump and continued for a few seconds after landing, allowing for comprehensive monitoring of the entire jumping process.

**Figure 1:**
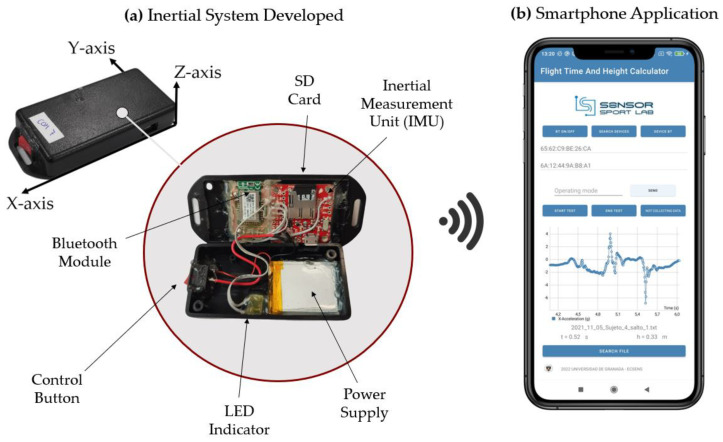

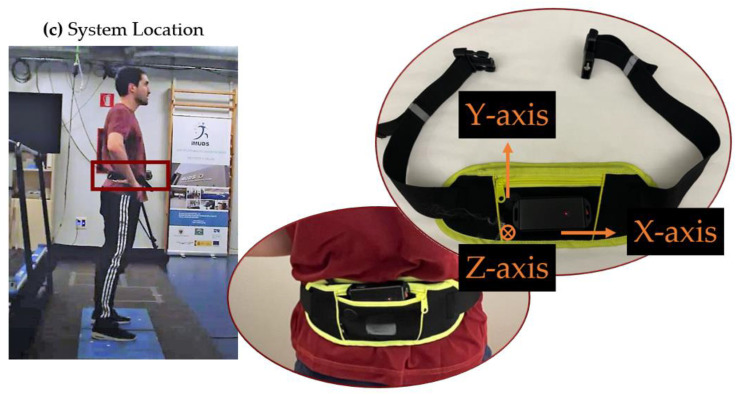
Overview of the developed inertial system and the custom smartphone application for the measurement of the flight time and reached height: (**a**) Inertial system developed both in open and closed cases; (**b**) Smartphone application designed for data communication and visualization purposes; (**c**) Placement of the system on the subject’s body.

The hardware of the developed system comprises an Inertial Measurement Unit, a Bluetooth module, a rechargeable power supply, and an SD card slot (see Figure 1a). The system is securely positioned on the lower back of the subject’s waist using a tight sport belt, as close as possible to the mass center [34,36,37,47,48] (see Figure 1c). The orientation of the IMU is depicted in Figure 1c, where the Y-axis corresponds to the vertical direction. The prototype utilizes the 9DoF Razor IMU MO (SparkFun Electronics, Niwot, CO, USA), which combines a SAMD21 microcontroller (Microchip Technologies Inc. Systems, Chandler, AZ, USA) with an MPU-9250 9DoF (Degree of Freedom) sensor (TDK InvenSense, San Jose, CA, USA), providing nine degrees of inertial measurement. The sensor includes a MEMS triple-axis gyro (ITG-3200) (TDK InvenSense, San Jose, CA, USA), triple-axis accelerometer (ADXL345) (Analog Devices, Wilmington, MA, USA), and triple-axis magnetometer (HMC5883L) (Honeywell, Charlotte, NC, USA). The ADXL345 accelerometer was configured within the range of ±16 g, where g represents the gravity acceleration. The acceleration was digitalized with a 16-bit analog-to-digital converter with a resolution of (1/2048) g, and a tolerance of 3%. The system also incorporates an SD card socket for data storage and a power supply module that monitors the Li-ion battery charge and regulates the power supply voltage. Wireless communication is facilitated by a BM78 Bluetooth module (Microchip Technologies Inc., Chandler, AZ, USA). An LED indicator is included in the device to display the system’s status. Consequently, the LED starts flashing when the power button is turned on, blinks quickly during the Bluetooth paring process, and blinks at a slower rate during measurements. To ensure extended operation during multiple training sessions, the system is equipped with a 450 mAh lithium-polymer battery, which offers sufficient autonomy as the average consumption of the system is approximately 50 mA. In terms of physical characteristics, the system weighs 47 g, and has dimensions of 80 × 40 × 15 mm, making it practically unnoticeable to the athlete.

To reduce the sampling time, only the acceleration components were monitored and directly transmitted via Bluetooth to the PC or smartphone, without storing them on an SD card. This approach allowed for a sampling frequency of 200 Hz, which is comparable to the sampling frequency of the reference system.

**Figure 2:**
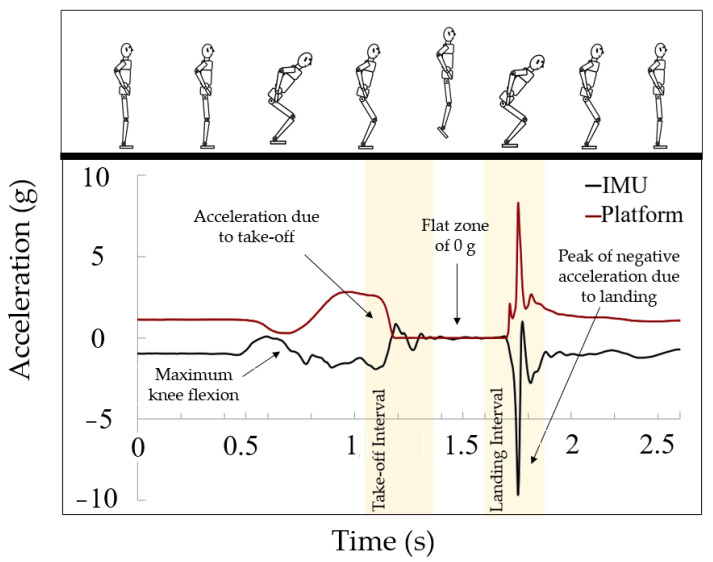
Experimental results of the jumping tests. Data reported by the inertial system (black line) and data provided by the force platform (red line).

The jumps were simultaneously monitored using both the proposed inertial system and the force platform, as depicted in Figure 2. To facilitate data analysis, the measurements obtained from both systems were synchronized by aligning the moment of maximum force recorded on the platform with the moment of highest acceleration registered by the portable system (in absolute value). This synchronization was easily achieved since the moment of highest acceleration during the jump corresponds to the moment when the force exerted on the platform is maximum.

In the case of the platform, it registers a resting acceleration value of 1 g, which corresponds to the subject’s weight. The flat area at 0 g indicates the absence of applied force, which corresponds to the flight phase. The data then show a sharp peak during the subject’s descent upon returning to the ground. In this case, the flight time can be obtained from the data provided by the platform, since the exact take-off and landing points can be accurately identified using proprietary software. Therefore, flight time can be obtained by subtracting both time values. In addition, the software can directly provide the jump height, which will be also compared and analyzed.

For the inertial system, the accelerometer data displays an acceleration value of −1 g during the resting periods, since it represents the gravitational force acting in the negative direction along the axis, i.e., towards the ground. Then, there is a slight drop due to knee bending in preparation for the jump, followed by a significant increase corresponding to the take-off. After that, the measurements stabilize around 0 g, signifying the airborne phase, ending with a prominent peak upon landing. Taking into account this data analysis, an algorithm has been developed to identify the key points of the jumps.

**Figure 3:**
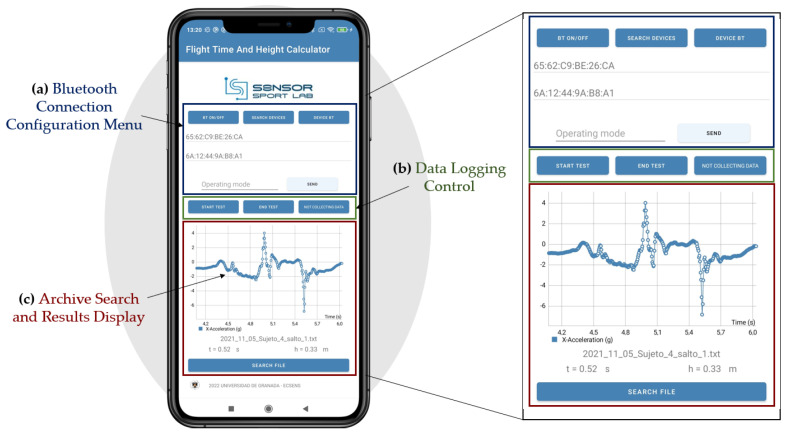
Overview of the custom smartphone application: (**a**) Bluetooth connection configuration menu; (**b**) Data logging control, used t initiate and terminate data acquisition and storage; (**c**) Archive search and results display.

The algorithm has been implemented as part of a smartphone application compatible with Android 12.0 (API level 31). It was developed on a Xiaomi RedMi Note 9 device using Android Studio (version 2020.3.1). By connecting the inertial device to the smartphone via Bluetooth, the user can start and stop the data collection process. The application also enables the storage of the acceleration measurements in a file along with the date and time of the test. The user interface of the application is depicted in Figure 3, where several menus can be observed. The Bluetooth communication menu is located at the top of the screen, allowing users to activate Bluetooth and select the desired device for connection. In the central part of the interface, there is a menu for data-acquisition control, allowing users to start and stop the test, indicating whether the device is in data-acquisition mode or not. Finally, at the bottom part of the interface, both numerical values and graphical representations of the acceleration over time are displayed, presenting the test results.

Before starting with the algorithm and data analysis, a pre-processing step must be performed. This pre-processing involves discarding the anomalous data, such as duplicate transmissions or time values outside the system’s operating scale. All these cases are easily detectable and treatable, ensuring the avoidance of potential failures in the measurement or reception of the information.

### 2.2. Flight Time and Jump Height Determination Algorithm

**Figure 4:**
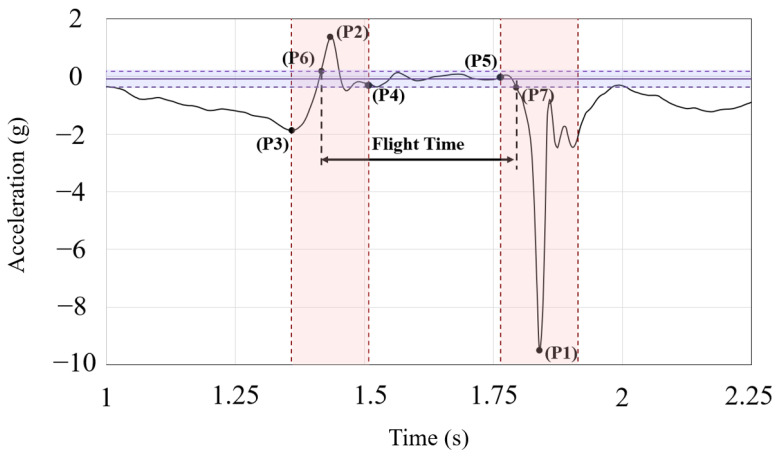
Explaining graph showing the time-of-flight calculation algorithm from the IMU measurements.

Once all the data were collected, stored, and pre-processed, they were analyzed to obtain the TOF and the height reached for each jump. For this purpose, the following algorithm is proposed and programmed into the smartphone application (Figure 4). The algorithm consists of 5 steps, which are described as follows:

Step 1: Delimitation of the intervals of interest

In this type of motion, the shape of the acceleration always exhibits a pattern similar to the one shown in Figure 4. The first step of the algorithm consists of detecting the locations of two crucial points:

- P1: This point is the absolute minimum among all the data collected by the inertial system, and corresponds to the descent to the ground after the jump.
- P2: This point represents the absolute maximum of the data and is associated with the moment when the upward impulse occurs during the jump.

Although these points will be close to the moments when the subject leaves the ground and lands on it again, they will not precisely coincide. However, they serve as reference points to estimate an interval within which they can be found. To determine the width of these intervals, another characteristic of this type of jump is considered. At the initial stage of the movement, point P3 can be identified, which corresponds to a minimum associated with the momentum acquisition through knee flexion. This will be the relative minimum closest to P2.

Therefore, we could define the intervals in which the take-off and landing points can be found, obtaining the half-interval measurement. These intervals are calculated as the time periods between points P2 and P3, and the centers of these intervals, which were determined in the previous step as P2 (take-off interval) and P1 (landing interval). In this way, a substantial amount of data is automatically filtered out, as it is based on the identification of characteristic points of this type of graph.

Step 2: Determination of the interval during which the subject is in the air

Throughout the jump, acceleration peaks are likely to occur during the preparation and return to the ground phases, while in the central zone, the subject remains suspended in the air, with an acceleration that approaches zero.

The next step consists of estimating the acceleration value associated with this interval, which can be accomplished using the points derived from step 1. The mean of the recorded values between points P4 and P5 is determined, along with its standard deviation. In this way, the subject can be considered to be in the air as long as the measured acceleration falls within the interval defined by this mean, with a tolerance of ±2 SD.

Although this scenario is not common, the possibility of an overlap phenomenon occurring in a jump characterized by a long preparation and minimal flight time has been considered. To increase the algorithm’s robustness, a code segment has been implemented to account for situations where the occurrence of P5 precedes that of P4. By reversing the role of these points in the calculation process, the rest of the code structure is maintained.

Step 3: Determination of the exact take-off and landing points

The intervals derived from steps 1 and 2 are used to precisely identify the take-off and landing points. For this purpose, within the take-off interval, the first value exceeding the threshold of the “floating acceleration” value is designated as the exact take-off point (P6), while within the landing interval, the first value that falls below this threshold is identified as the exact landing point (P7).

A minor modification is implemented to improve the accuracy of the system by using linear interpolation to find the exact time value of the jump at which these boundary-crossing conditions occur within the interval. Otherwise, a small error would be introduced, considering that measurements are taken every 10 ms, which can be approximately 3% of additional error within the range of values obtained from the trials.

Step 4: Calculation of the flight time

The difference between the times at which the take-off and landing points occur is the value of the TOF.

Step 5: Determination of the reached height

Although the data analysis algorithm can be considered as completed in the previous step with the determination of the flight time, the calculation of the reached height (*h*) has been included to provide the athletes with additional information to enhance their techniques. For this purpose, a physical model using the time-of-flight time (TOF) as a parameter has been employed (1).

(1) $$h=\frac{1}{2}\cdot g\cdot {\left(\frac{\mathrm{TOF}}{2}\right)}^{2}$$

## 3. Results

Table 1, Table 2, Table 3 and Table 4 show the results obtained from our IMU-based system and the gold standard (platform) for all tests, including both the TOF and height measurements, respectively. Relative errors have also been calculated, showing low values that align with the accelerometer tolerance (3%), thus pointing out an excellent agreement between the gold standard and the proposed system.

**Figure 5:**
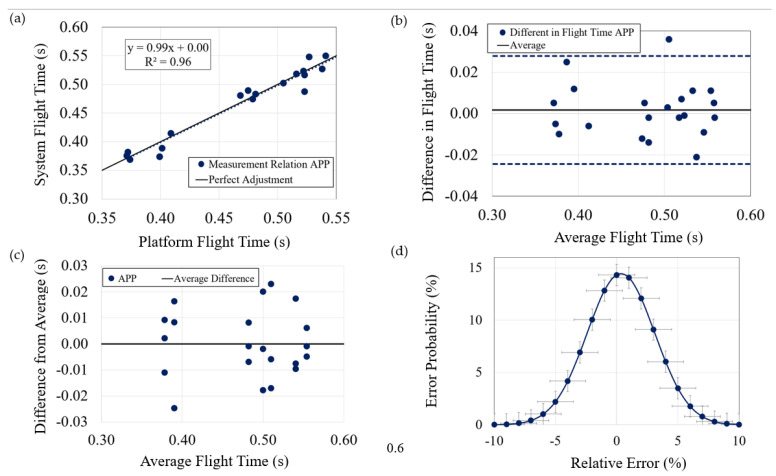
Statistical analysis of data (flight time): (**a**) Data correlation; (**b**) Deviation concerning average of both systems; (**c**) Deviation for each subject; (**d**) Gauss bell of flight time error.

The collected data have been processed, and a statistical analysis was conducted to assess the level of agreement between the data obtained from the force platform and the developed system. The measurement errors were determined as the difference between the TOF values reported by the inertial system and the force platform. Regarding the level of agreement, the relationship between the chosen reference and the values of the developed system was examined. This analysis provides an indication of the accuracy of the measurements. To achieve this, the corresponding linear regression was calculated (Figure 5a), which indicates both the linearity fit of the data and the level of agreement with the expected values. In this case, an excellent agreement was obtained, with a linear regression equation of 0.99x + 0.00 and an $R^{2}$ factor of 0.96.

The next aspect to consider is the variation of the difference between the measurements obtained from the inertial system and the platform, in relation to their average for each test (Figure 5b). For the system to be considered reliable, this difference should fall within the range of ±2 SD. As can be observed in Figure 5b, the majority of cases exhibit differences that lie within this range, with only a few cases showing minimal deviation (a maximum of 12 ms) from this margin.

The final aspect to assess is the system’s repeatability. For this purpose, the data from the three jumps performed by each subject measured with the developed system were studied separately, considering that the flight time should be similar on each attempt if conducted under the same conditions (Figure 5c). The maximum deviation observed is only approximately 24.67 ms, which represents about a mere 5% error. Hence, it can be concluded that repetitive tests can be conducted successfully. Regarding the errors, the average of all cases considering the sign is (0.4 ± 3)%, which indicates that there is practically no systematic error. If error percentages are taken in absolute value, the average becomes (2.1 ± 1.8)%, which is a remarkably small value. In addition, Figure 5d shows the Gaussian distribution of the errors, revealing the system’s high accuracy, as the probability of an error exceeding 5% is approximately 10.4%.

**Figure 6:**
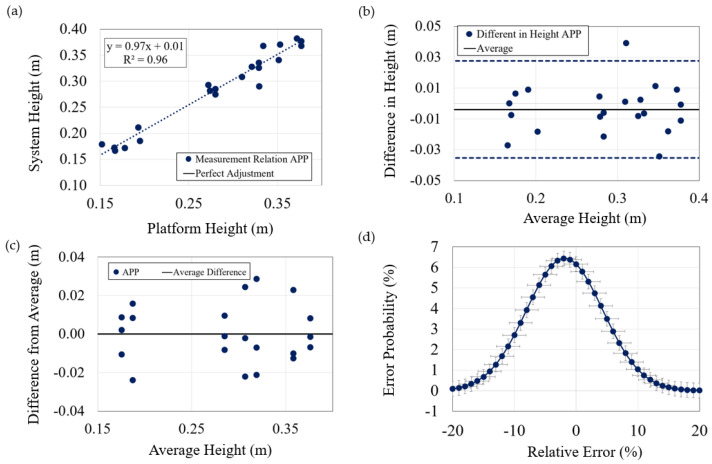
Statistical analysis of data (height). Analysis carried out in the same way as for the flight time, including: (**a**) Data relation; (**b**) Deviation concerning average of both systems; (**c**) Deviation of each subject; (**d**) Gauss bell of height error.

Similarly, the data and results for the case of jump height measurements were analyzed (Figure 6). The obtained linear regression was 0.97x + 0.01, with an $R^{2}$ factor of 0.96. The variation of the difference between the measurements obtained from the system and the platform, in relation to their average for each test, also falls within the range of ±2 SD, with the exception of one value exhibiting a deviation of 0.011 m from the margin. Additionally, the maximum difference between the average of the three attempts of a subject and the measurement of each individual attempt is only 0.028 m. In terms of errors, when considering their absolute values, they result in (4.57 ± 4.48)% (~0.013 ± 0.13 m), while they are (−1.84 ± 6.2)% (~−0.005 ± 0.018 m) when considering the sign. Both cases still provide very low values. Finally, the probability of encountering an error greater than 10% is only 4.69%.

## 4. Discussion

In light of the obtained results, it can be noted that the measurements obtained from the proposed system achieve a high level of accuracy in comparison with the gold standard. This is supported by the small measurement errors observed, which amount to approximately 0.35% for flight time and around −1.84% for height. Furthermore, the repeatability of the measurements is demonstrated by the consistency of the subject’s marks across their attempts. It is worth noting that the error estimated for height is slightly higher than that for the TOF. This can be attributed to the fact that the jump time value is used as a parameter in the physical model employed for the calculation. Consequently, any component due to the error in the jump time would be added to the overall error of the model itself. Even in this case, the error remains sufficiently low to consider the system reliable. In addition, regardless of the test jump performed, the inertial system showed virtually zero systematic bias and a low random error.

When comparing the developed system with existing systems currently available in the literature that are based on accelerometers and video systems (Table 5), it can be seen that the results obtained in this study are suitable and, furthermore, it fulfils all the aforementioned requirements: It is low cost, has minimal impact on the test subject due to its reduced dimensions and lightweight design, and it is completely portable facilitated by Bluetooth communication and the associated application for data processing.

Focusing on the systems with higher sampling frequencies, it can be concluded that most of them present greater deviations in the measurements (x-BIMU Bluetooth Kit, Myotest accelerometric system, IMU Push 2.0, KineJump), or they require a connection to a PC via USB for data acquisition (KineJump, YEI 3-Space sensors). The only system that demonstrates comparable performance (NGIMU, x-io Technologies) presents the disadvantage of requiring an external computer for data processing, which may limit its applicability for on-site and real-time data analysis.

## 5. Conclusions

In this work, a lightweight and portable electronic system designed for measuring and processing data from jumping tests is presented, demonstrating its feasibility for monitoring training sessions. Several tests involving different subjects were performed, obtaining satisfactory results, with low measurement error and high repeatability. An Android application was developed to provide a user-friendly interface, facilitating data interpretation. The system offers reliable and accurate measurements of flight time and jump height, while its small size and complete portability make it desirable for use in training sessions. After all the conducted tests, the system exhibited minimal error of just 0.35% in the case of the flight time and −1.84% for the height measurement. Hence, this system proves suitable for outdoor training sessions, enabling in-site and in real-time performance monitoring of both high-level athletes and amateurs.

## Figures and Tables

**Table 1:** Flight time measurements (s).

	Platform			Inertial System		
	Jump 1	Jump 2	Jump 3	Jump 1	Jump 2	Jump 3
Subject 1	0.51	0.47	0.52	0.50	0.48	0.52
Subject 2	0.37	0.41	0.40	0.38	0.42	0.37
Subject 3	0.48	0.48	0.48	0.47	0.49	0.48
Subject 4	0.52	0.53	0.54	0.52	0.55	0.55
Subject 5	0.52	0.52	0.54	0.52	0.49	0.53
Subject 6	0.37	0.37	0.40	0.38	0.37	0.39
Subject 7	0.56	0.56	0.56	0.56	0.55	0.56

**Table 2:** Relative errors of the inertial system measurements concerning those of the platform (reference) in terms of flight time (%).

	Jump 1	Jump 2	Jump 3
Subject 1	0.59	−2.56	−0.39
Subject 2	−2.69	−1.47	6.47
Subject 3	1.04	−2.95	−0.42
Subject 4	−0.19	−3.99	−1.66
Subject 5	1.34	6.88	2.05
Subject 6	−1.35	1.34	2.99
Subject 7	0.89	1.97	−0.36

**Table 3:** Height measurements (m).

	Platform			Inertial System		
	Jump 1	Jump 2	Jump 3	Jump 1	Jump 2	Jump 3
Subject 1	0.31	0.27	0.32	0.31	0.28	0.33
Subject 2	0.15	0.19	0.18	0.18	0.21	0.17
Subject 3	0.28	0.27	0.28	0.28	0.29	0.29
Subject 4	0.33	0.33	0.35	0.34	0.37	0.37
Subject 5	0.33	0.33	0.35	0.33	0.29	0.34
Subject 6	0.17	0.17	0.195	0.17	0.17	0.19
Subject 7	0.38	0.38	0.37	0.38	0.37	0.38

**Table 4:** Height errors (%).

	Jump 1	Jump 2	Jump 3
Subject 1	0.31	−3.08	2.47
Subject 2	17.69	9.39	3.67
Subject 3	1.63	−7.77	−2.14
Subject 4	−1.92	−10.2	−5.05
Subject 5	0.79	11.9	3.28
Subject 6	−4.40	0.05	4.62
Subject 7	−0.16	2.35	−2.97

**Table 5:** Comparison of current systems.

System	Sampling Frequency	Data Sending/Processing Method	Mean Bias	Acceleration Range	Correlation Coefficient
KineJump [34]	640 Hz	Data to PC via USB	−11.7 (±5.1) cm	±6 g	0.85
IMU (Sensorize, Rome, Italy) [37]	100 Hz	Data to PC via Bluetooth	0.6 cm	±6 g	0.87
iPhone s5 (APP) [40]	120 Hz	Video Recording	1.1 (±0.5) cm	-	0.99
YEI 3-Space sensors (Yost Engineering, Portsmouth) [35]	300 Hz	Data in flash memory PC via USB	0.3 (±3.2) cm	-	0.98
IMU Push 2.0 Strength Inc., Toronto, Canada [36]	200 Hz	Bluetooth Connection to APP	+8 cm	-	0.89
NGIMU, x-io Technologies Limited [39]	400 Hz	Treatment with Matlab (PC)	−0.1 cm	±16 g	0.97
Myotest accelerometric system (Myotest SA, Sion, Switzerland) [33]	500 Hz	-	7.24 (±2.82) cm	±8 g	0.98
x-BIMU Bluetooth Kit (x-io Technologies Limited, UK) [49]	256 Hz	-	5.5 cm	±16 g	0.90
This system	200 Hz	Data to APP via Bluetooth	−0.5 cm	±16 g	0.96

The technique used for the jumps was the CMJ (counter-movement jump).

## Data Availability

The data presented in this study are available on request from the corresponding author. The data are not publicly available due to the amount of data.
